# COVID-19 Epidemic in Bangladesh among Rural and Urban Residents: An Online Cross-Sectional Survey of Knowledge, Attitudes, and Practices

**DOI:** 10.3390/epidemiologia2010001

**Published:** 2020-12-22

**Authors:** Md. Siddikur Rahman, Ajlina Karamehic-Muratovic, Miftahuzzannat Amrin, Arman Hossain Chowdhury, Md. Selim Mondol, Ubydul Haque, Parveen Ali

**Affiliations:** 1 Department of Statistics, Begum Rokeya University, Rangpur 5400, Bangladesh; miftahuzzannatamrin@gmail.com (M.A.); armanhossain1710044@gmail.com (A.H.C.); selimmondol2@gmail.com (M.S.M.); 2 Department of Sociology and Anthropology, Saint Louis University, St. Louis, MO 63108, USA; ajlina.karamehicmuratovic@slu.edu; 3 Department of Biostatistics and Epidemiology, University of North Texas Health Science Center, Fort Worth, TX 76107, USA; 4 Health Sciences School, The University of Sheffield, Barber House Annexe, 3a Clarkehouse Road, Sheffield S10 2LA, UK; parveen.ali@sheffield.ac.uk

**Keywords:** COVID-19, Bangladesh, prevention, control

## Abstract

As other nations around the world, Bangladesh is facing enormous challenges with the novel coronavirus (COVID-19) epidemic. To design a prevention and control strategy for this new infectious disease, it is essential to first understand people’s knowledge, attitudes, and practices (KAP) regarding COVID-19. This study sought to determine KAP among rural and urban residents as well as predictors of preventive practices associated with COVID-19 in Bangladesh. A social media-based (Facebook) cross-sectional survey was conducted to explore these variables among Bangladeshi adults. Of 1520 respondents who completed the questionnaire, low level of good or sufficient knowledge of COVID-19 (70.8%) and practices associated with COVID-19 (73.8%) were found. Despite the low level of knowledge and practices, respondents’ attitude (78.9%) towards COVID-19 was relatively high. Results suggest that compared to urban, rural residents are at a particularly high risk of COVID-19 because they were found to have significantly lower knowledge (*p* = 0.001) and practice levels (*p* = 0.002) than were urban residents. Multivariable logistic regression analysis identified gender, education, knowledge of COVID-19 transmission, signs and symptoms, and sources of information as factors significantly associated with preventive practices against COVID-19. Further attention and effort should be directed toward increasing both knowledge and practices targeting the general population in Bangladesh, particularly the rural and less educated residents. Findings from this study provide baseline data that can be used to promote integrated awareness of and effective health education programs about COVID-19 prevention and control strategies in Bangladesh, and similar COVID-19 endemic countries.

## 1. Introduction

The novel Coronavirus (COVID-19) pandemic is a major global health threat of the twenty-first century and was first detected on 31 December 2019 in Wuhan, China [1,2]. WHO declared COVID-19 a global emergency on 30 January 2020, and labelled it a pandemic on 11 March 2020. This new virus is structurally similar to severe acute respiratory syndrome (SARS) and the Middle East respiratory syndrome (MERS) [3,4], but does not seem to be as deadly as other coronaviruses including SARS and MERS [5]. During the 2002–2003 SARS outbreak, a total of 8098 infected cases and 774 deaths were reported [5]. In the later outbreak of MERS and since 2012, a total of 2494 infected cases and 858 deaths were reported [3,6]. COVID-19 has, however, surpassed the earlier outbreaks of coronaviruses and is more transmissible than both SARS and MERS [7]. As of 16 December 2020, 190 countries, including Bangladesh, have confirmed more than 73.5 million COVID-19 cases and 1.64 million deaths globally [8].

COVID-19 has exposed large gaps in public health preparedness and response for infectious disease threats and outbreaks in South Asia, including Bangladesh. The lack of resilient public health surveillance system is particularly evident [9]. Bangladesh is one of the poorest and most densely populated countries in the world, with over 160 million inhabitants [10]. The first COVID-19 case in Bangladesh was identified by the Institute of Epidemiology, Disease Control and Research (IEDCR), on 7 March 2020 [11]. The number of infected cases began to rise on 9 March 2020, and as of 16 December 2020, there were 494,209 infected cases and 7129 deaths reported in Bangladesh [8,11]. According to the COVID-19 risk and vulnerability index, Bangladesh ranked 30th in the globe in the number of individuals affected [12]. The COVID-19 outbreak has already spread across all 64 districts in the respective nation (IEDCR, 2020). However, questions remain regarding the actual number of reported cases and shortage of testing facilities [13]. Bangladesh is facing an unprecedented challenge to protect against COVID-19 because of its high population density and fragile healthcare system.

With vaccines starting to become available in several countries to select residents such as the elderly and health workers, positive individual and communal actions continue to be major ways by which to minimize the transmission of the virus and potentially save lives [14]. WHO has outlined public health and social measures that are useful for slowing or stopping the spread of COVID-19 at local, regional, national, and international level. These include, but are not limited to, practicing social distance, regular hand wash, avoid touching one’s face, smoking cessation, and avoiding other activities that weaken the lungs (WHO 2020). A limited number of knowledge, attitudes, and practices (KAP) studies have been conducted in Bangladesh during previous epidemics. For instance, a study conducted in Bangladesh during the dengue epidemic in 2019 found that good knowledge and attitudes were significantly associated with good dengue prevention practices [15]. Battling the COVID-19 pandemic is a lengthy process and measures to raise the general population’s knowledge and implementation of recommended health practices are some of the best approaches to combat COVID- 19 [13,16]. Adopting individual level prevention strategies depends on people’s KAPs regarding the health threat, especially in infectious disease contexts [17].

A KAP survey is an effective tool for the management of infectious disease during outbreak and recovery stages [15]. This survey among at-risk populations is useful to provide critical information to guide response and recovery efforts, health education, and social mobilization during epidemics [15]. Bangladesh fears a coronavirus crisis as cases could be increasing due to higher testing rates, testing equipment becoming more available (whereby earlier in the pandemic testing was not readily available), infections increasing, or a combination thereof. Thus, there is a growing concern about the importance of health behaviors and attitudes towards the virus, and studies that address KAP in Bangladesh can contribute to prevention of further spread. Densely populated and overcrowded countries such as Bangladesh have the potential to become disease hotspots with active transmission of agents to large communities [13].

Under such an alarming situation, little is known about the status regarding COVID-19 KAP among Bangladeshi residents. To facilitate outbreak management of COVID-19, an understanding of the public’s awareness related to COVID-19 signs and symptoms, and transmission modes and treatments/prevention/control strategies towards COVID-19 is urgently needed. Therefore, this study aimed to investigate KAP and determinants of preventive practices related to COVID-19 outbreak among rural and urban residents in Bangladesh. This study provides a glimpse of the overall public health preparedness and a scientific basis for preventing and controlling the epidemic in Bangladesh and other endemic countries around the world. The following research questions were addressed in this study: (1) Can differences in people’s KAP towards COVID-19 be explained by their socio-demographic characteristics? (2) Do participants’ demographics, knowledge, and attitudes about COVID-19 signs and symptoms, transmission, and/or preventive measures contribute to better preventive practices?

## 2. Materials and Methods

### 2.1. Study Design and Respondents

A cross-sectional, population-based online survey was conducted via Facebook between 15 March and 15 April 2020, during the onset of COVID-19 outbreak in Bangladesh. The study respondents were of Bangladeshi nationality, aged 18 years and above, and regular Internet and Facebook users. Detailed study framework is given in the Supplementary Materials (Figure S1).

### 2.2. Instrument

The questionnaire used in the study was developed based on the available information from the World Health Organization, the Center for Disease Control and Prevention (USA), and the Ministry of Health and Family Welfare (Bangladesh) [18,19,20]. Furthermore, the questionnaire was validated by a panel of experts consisting of an epidemiologist, microbiologist, health educator, and medical statistician. The questionnaire was translated into the native language “Bangla” by the author and checked and validated for cultural appropriateness. For the questionnaire validity test, a pilot study was conducted where the overall Cronbach’s Alpha coefficient of KAP domains was 0.70; for each domain, coefficients were 0.74, 0.66, and 0.56, respectively (Table S1). The questionnaire included 46 items divided into four sections: (i) demographics, (ii) knowledge, (iii) attitudes, and (iv) practices associated with COVID-19. Demographic characteristics included age, gender, marital status, educational background, and income. The knowledge section consisted of 20 items and was aimed at accessing and evaluating the general knowledge of respondents about the possible spread, transmission, signs and symptoms, risk factors, and prevention of COVID-19. In the attitude section, four questions assessed the behavioral perception of prevention. Twelve questions on practices evaluated the actual compliance and uptake of various preventive measures.

### 2.3. Data Collection

The online survey was conducted among 1520 respondents (911 rural and 609 urban) in Bangladesh. Using the authors’ networks and various Facebook groups associated with several rural and urban regions of Bangladesh, the week following the outbreak in Bangladesh, respondents were invited via Facebook to complete the survey. The link also provided an option for respondents to invite their family and friends to take part in the study [15].

Social networking sites such as Facebook provide a useful platform for research especially in current circumstances when it is not feasible to do a community-based national sampling survey. Written consent was forwarded to the target respondents via Facebook through private and group chats. Respondents who gave consent to participate were then sent a link to the questionnaire and accompanying instructions to complete it. Respondents were assured that the information they provide would be kept confidential.

### 2.4. Sampling

Sample size was calculated using RaoSoft online sample size calculator [21]. Since the KAP level of the study population is unknown, the most statistically conservative response distribution was assumed to be 50% [22], confidence interval was set at 95% with a margin of error 2.5%, and the calculated sample size was 1520. However, responses to the online survey continued to be collected until the online survey portal closed.

### 2.5. Data Management and Statistical Analysis

For the knowledge questions, incorrect or uncertain (do not know) responses were assigned a 0 score, while 1 was assigned for choosing the correct answer. The maximum total knowledge score was 20. A Likert-type scale was used to assess agreement or disagreement with an attitudinal statement, where 5 represented strongly agree, 4 was agree, 3 was not sure, 2 was disagree, and 1 was strongly disagree. The maximum total attitude score was 20 and the minimum score was 12. For practice sections, a score of 1 was assigned if the respondent selected an answer reflecting good practice and 0 was assigned to the answer reflecting poor practice. Respondents’ KAP levels were measured using a scoring system, where scores ≥80% were classified as having good KAP, while those with scores <80% were considered as having poor KAP [15]. As analytical tool, Pearson’s chi-square test was used to examine the associations between independent and dependent variables. The odds ratio (OR) and its 95% confidence interval (CI) were also reported (Table S2). Multivariable analysis using a forward conditional stepwise logistic regression model was used to identify significant predictors associated with preventive practices against COVID-19 considering variables with *p*-values less than or equal to 0.2 (Table S3). Data analysis was performed using the IBM SPSS Statistics version 24.0 for Windows (IBM Corp., Armonk, NY, USA) and RStudio (Version 1.1.456—© 2020–2018 RStudio, Inc., Boston, MA, USA).

## 3. Results

### 3.1. Socio-Demographic Characteristics

From the 1520 respondents surveyed, 40.1% were residents of urban and 59.9% were residents of rural areas (Table 1). The most frequent age group was <30 years, with a mean age of 30.1 (*SD* = 6.1). The majority of the study respondents were male (62.1%), unmarried (74.2%), and had higher secondary level of education (76.4%) (Table 1). The monthly income category of > BDT 30,000 was the highest (50.4%) among urban residents (Table 1). A chi-square test for independence indicated that all demographic variables (Table 1) were significantly different between urban and rural residents (*p* < 0.01).

### 3.2. Correct Knowledge of Respondents on COVID-19

Most of the respondents could correctly identify transmission modes of COVID-19, such as touching and shaking hands with an infected person (88.5%), use of objects used by an infected person (77.1%), and close contact (72.1%), with significantly higher correct responses from urban respondents compared to rural respondents (*p* < 0.01) (Table 2). Seventy-seven percent of the rural respondents and 78.7% of the urban respondents identified correctly the COVID-19 transmission mode to be person-person transmission (Table 2). Only 58.4% of the respondents indicated that COVID-19 could be transmitted through contact with respiratory droplets with significantly higher responses from urban respondents compared to rural respondents (*p* < 0.01) (Table 2). Few respondents (31.7%) knew that COVID-19 can be transmitted through the sexual route which was not significantly different between urban and rural respondents (Table 2). Most of the respondents correctly perceived that fever (96%), dry cough (87.5%), and shortness of breath/breathing difficulties (81.5%) are the main signs and symptoms of COVID-19 (Table 2). Most respondents correctly identified tiredness (52.5%) and diarrhea (51.4%) as signs and symptoms of COVID-19. However, nasal congestion (31.8%) was the least frequent symptom of the disease correctly identified by the respondents, followed by runny nose (35%) and sore throat (44.3%) (Table 2). Regarding knowledge scores, only 25% of the rural respondents had good knowledge about general signs and symptoms, transmission modes, and treatment/preventions, with a significantly higher knowledge among urban respondents (*p* < 0.01) (Table 2).

### 3.3. Attitudes of the Respondents towards COVID-19

The majority of the respondents reported positive attitudes and strongly agreed on the importance of measures towards COVID-19, such as lock-down (74.3%), followed by maintaining personal hygiene (65.7%), home quarantine (52%), isolation and treatment of infected people (51.3%), with significantly higher (*p* < 0.01) positive attitude among urban respondents compared to rural respondents (Table 3). Regarding attitude scores, 84.9% of the urban respondents had a significantly higher positive attitude towards COVID-19 (*p* < 0.01) compared to rural respondents (Table 3).

### 3.4. Good Preventive Practices against COVID-19

The majority of the respondents (88.8%) identified staying away from the infected person as a good preventive practice against COVID-19 infection, followed by frequent hand washing using hand sanitizer (83.2%), avoiding touching nose, mouth, and eyes (78.2%), using a face mask (77.6%), practicing self-isolation/home quarantine (88%), and avoiding hugging (75.7%) (Table 4). Significantly higher responses about preventive practices of COVID-19 infection such as practicing respiratory hygiene (57.7%) and maintaining social distance (min 1 m) (56.4%) were more common among urban respondents compared to rural respondents (*p* < 0.01), except for avoiding handshake (50.3%) (Table 4). Ensuring sufficient food stock (33.4%) and avoiding a visit to public places (42.3%) were the least frequent practices mentioned by the respondents (Table 4). Regarding overall practice scores, only 32.5% of the urban respondents had good preventive practices against COVID-19, though these were significantly higher compared to rural respondents (22%) (*p* < 0.01) (Table 4).

### 3.5. Sources of Information on COVID-19

**Figure 1:**
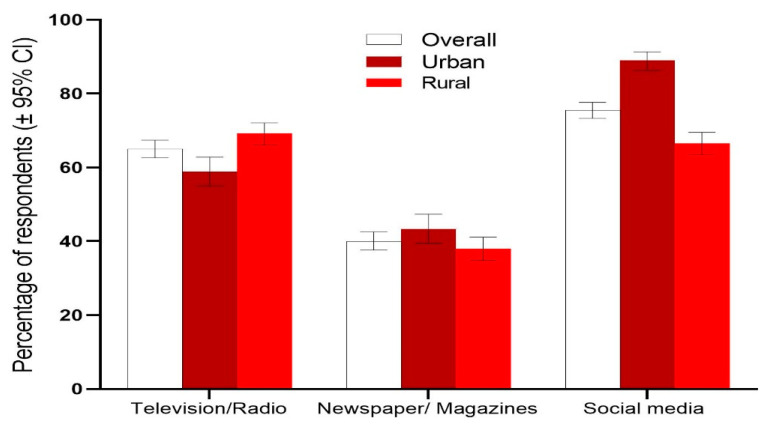
Sources of information on COVID-19. Error bars represent 95% confidence intervals.

Majority of the respondents (76%) had heard of COVID-19 through social media (Facebook/Twitter/YouTube/Instagram), followed by television/radio (65%), and newspapers/magazines (40%), with significantly higher responses from urban respondents (*p* < 0.05). Social media was reported as the common source of information among both urban (89%) and rural (66.5%) respondents (Figure 1).

### 3.6. Analysis of Demographic Factors, Knowledge, and Attitudes Associated with Preventive Practices against COVID-19

**Figure 2:**
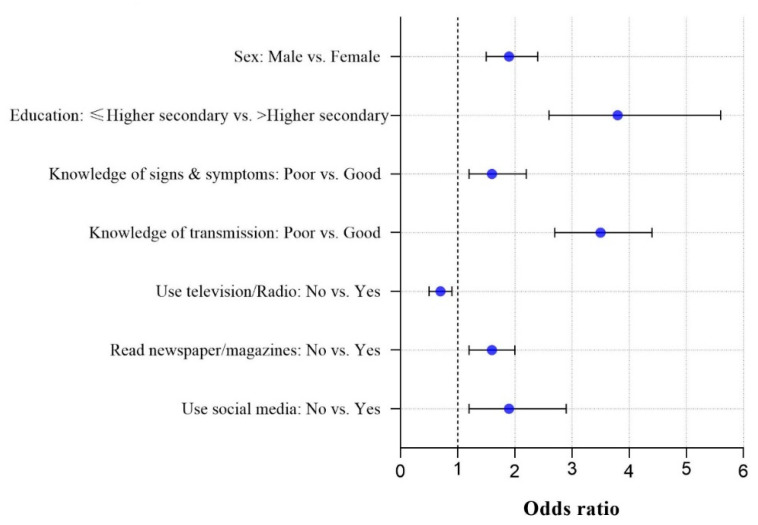
Significant factors associated with poor prevention practices regarding COVID-19 in Bangladesh.

Male respondents were 1.9 times more likely to have poor COVID-19 prevention practices compared to female respondents (Figure 2). Those respondents who had higher secondary or lower (college) education were 3.8 times more likely to have poor COVID-19 practices compared to those who had more than higher secondary level education (tertiary education). Respondents who had poor knowledge of transmission of COVID-19 had 3.5 times poorer COVID-19 prevention practice compared to respondents who had good knowledge of transmission. In addition, increased odds of having poor COVID-19 prevention practices were identified among respondents with poor knowledge compared to respondents with good knowledge associated with signs and symptoms of COVID-19. Respondents who did not use television/radio had 0.7 times poorer COVID-19 prevention practices than respondents who used this media. Daily newspaper/magazine non-readers were 1.6 times more likely to have poor COVID-19 prevention practice compared to readers. Finally, non-users of social media were 1.9 times more likely to have poor COVID-19 prevention practices compared to users (Figure 2).

## 4. Discussion

This study was conducted during the onset of COVID-19 pandemic in Bangladesh and elsewhere. Overall, 70% of the respondents surveyed in Bangladesh had poor knowledge and preventive practices regarding COVID-19, even though most of the respondents (more than 60%) reported good attitude towards COVID-19. This latter finding is encouraging and offers opportunity for intervention. Male and less educated respondents who also have poor knowledge on signs and symptoms and transmission regarding COVID-19 reported poor prevention practices towards COVID-19. Several studies also found that in Bangladesh more than half of the respondents reported “good knowledge” of COVID-19, with age and education having a significant impact on knowledge and prevention practices of COVID-19 [13,23,24]. Another study in China found that age, gender, and education were influencing factors of COVID-19 knowledge [25]. In contrast, a study conducted by Zhong et al. showed that the overall correct rate of COVID-19 knowledge was 90% [1], which was much higher than that of our study; the difference might be due to the higher education levels of the sample compared to our study. Therefore, relevant health education can help improve KAP level of the public, especially those with lower educational background.

Urban respondents reported comparatively better KAP than rural respondents; the difference may be due to better access to education, internet facilities, communication strategies, and health facilities which was reflected in the respondents’ level of KAP. For example, we found that more than 90% of the urban participants had higher than secondary school level of education compared to rural participants (66.1%). Higher socio-economic conditions and internet usage was also higher among urban participants (Table 1). Low literacy rate, internet facilities, and poor socioeconomic conditions were likely key factors resulting in insufficient KAP regarding COVID-19 among rural participants [25]. This study also identified significant determinants of prevention practices towards the pandemic in Bangladesh. These findings are useful for public health policymakers and health workers to identify target populations for COVID-19 prevention and health education. KAP surveys are a useful tool to determine effective evidence-based prevention and control strategies through changing poor KAP [26].

We found that most respondents knew that COVID-19 is a global pandemic and social media was found to be the most popular and frequently used source of information among both urban and rural communities. These findings suggest that people are more interested in receiving news and information about COVID-19 through social media. This finding can be used by the Bangladesh Ministry of Health to promote population-based awareness regarding COVID-19 through social media. As there are currently no completely effective vaccines against COVID-19, prevention and management remain the best and only way to tackle this deadly disease. Public awareness of COVID-19 through social media and mass media is a crucial factor in protecting against this disease. At the same time, however, it is important to ensure that correct information on COVID-19 is spread via both social and mass media, provided that misinformation about COVID-19 continues to dominate social media [27]. Consuming credible and heterogeneous rather than polarized and siloed health information and news via social media is particularly important because it has a significant effect on one’s healthcare decisions and outcomes.

Online-based mental health intervention programs are strongly recommended as a way of promoting more reliable and authentic information about COVID-19, as well as making available possible telemedicine care, as suggested in related research papers [28,29,30]. Both government and non-government agencies need to educate local communities about the protection and safety measures against COVID-19. Early and quick detection measures using emerging technologies can be applied to stop the transmission of COVID-19 [31]. The diagnostic facilities of Bangladesh should be improved across the country, particularly among vulnerable communities, such as older adults, the poor, minorities, the homeless, and those with pre-existing mental health disorders [32]. Further specific to Bangladesh, vulnerable communities include those who live in remote rural areas, the poor, children, and adolescents. As suggested by Rajkumar (2020), cases such as these require “close collaboration between psychiatrists and specialties from other branch of medicine, as well as with local authorities and health workers in the community” [32].

## 5. Study Limitations

This study had some limitations connected to the interpretation of results because COVID-19 is a novel coronavirus and there is not enough prior research to compare the results to. Likewise, caution should be exercised in generalizing the study to a national population, provided the limited sample size.

## 6. Conclusions and Recommendations

Above all, the attitude of Bangladeshi urban and rural residents is positive, but the knowledge and preventive practices related to COVID-19 need to be improved. There is an urgent need for building awareness programs targeting the unhealthy behaviors of rural and urban residents in Bangladesh. For this, social mobilization and communication programs should be developed. Since most of the respondents use social media and electronic media, rigorous and targeted campaigns by public health authorities through social, electronic, and print media can ultimately play a role in improving knowledge and control measures regarding COVID-19 by disseminating validated health information. As the global threat of COVID-19 continues to escalate, greater efforts through an interdisciplinary approach involving community participation, media, government, and educational programs regarding COVID-19 should be advocated to control the pandemic. This study provides useful information for COVID-19 control and prevention which is specific to Bangladesh, and suggests that routine KAP investigation can be an effective monitoring tool to control the spread of COVID-19.

## Figures and Tables

**Table 1:** Socio-demographic characteristics of study respondents (*n* = 1520) in Bangladesh.

	Rural		Urban			
Variables	*n*	% (95% C.I.)	*n*	% (95% C.I.)	Total *n* (%)	*p*-Value
Total respondents	911	59.9	609	40.1	1520 (100.0)	
Age group (years)						<0.01
<30	398	43.7 (40.5–46.9)	327	53.7 (49.7–57.6)	725 (47.7)	
30–40	213	23.4 (20.7–26.2)	206	33.8 (30.2–37.7)	419 (27.6)	
>40	300	32.9 (29.9–36.0)	76	12.5 (10.0–15.3)	376 (24.7)	
Sex						<0.01 *
Female	304	33.4 (30.4–36.5)	271	45.5 (40.6–48.5)	575 (37.8)	
Male	607	66.6 (63.5–69.6)	338	55.5 (51.5–59.4)	945 (62.1)	
Education						<0.01 *
≤ higher secondary	309	33.9 (30.9–37.0)	49	8.0 (6.1–10.4)	358 (23.6)	
> higher secondary	602	66.1 (63.0–69.1)	560	92.0 (89.6–93.9)	1162 (76.4)	
Marital status						<0.01
Unmarried	595	65.3 (62.2–68.4)	534	87.7 (84.9–90.1)	1129 (74.2)	
Married	311	34.1 (31.1–37.3)	75	12.3 (9.9–15.1)	386 (25.3)	
Divorced/Separated	5	0.5 (0.2–1.2)	N/A	N/A	5 (0.3)	
Monthly family income (BDT)						<0.01
<10,000	288	31.6 (28.7–34.7)	52	8.5 (6.5–11.0)	340 (22.3)	
10,000–20,000	247	27.1 (24.3–30.1)	104	17.1 (14.2–20.2)	351 (23.0)	
20,000–30,000	196	21.5 (18.9–24.3)	146	24.0 (20.7–27.5)	342 (22.5)	
>30,000	180	19.8 (17.3–22.4)	307	50.4 (46.4–54.4)	487 (32.0)	
COVID-19 is a global pandemic						0.215 *
No	13	1.4 (0.8–2.4)	4	0.7 (0.2–1.6)	17 (1.1)	
Yes	898	98.6 (97.6–99.2)	605	99.3 (98.4–99.8)	1503 (98.8)	
Use internet to learn about COVID-19						<0.01 *
No	136	14.9 (12.7–17.4)	28	4.6 (3.1–6.5)	164 (10.7)	
Yes	775	85.1 (82.6–87.3)	581	95.4 (93.5–96.5)	1356 (89.2)	

All *p*-values are based on a Chi-square test of numbers in urban and rural categories except those indicated by an asterisk (*) which are based on Fisher’s exact test. (BDT) Bangladeshi Taka.

**Table 2:** Correct knowledge/awareness about COVID-19 pandemic among urban and rural respondents of Bangladesh (*n* = 1520).

Knowledge Items	Rural		Urban			
	*n*	% (95% C.I)	*n*	% (95% C.I.)	Total *n* (%)	*p*-Value
Total respondents	911	59.9	609	40.1	1520 (100.0)	
Transmission modes						
k1. Contact with respiratory droplets	483	53.0 (49.8–56.2)	405	66.5 (62.7–70.2)	888 (58.4)	<0.01 *
k2. Touching and shaking hands with an infected person	781	85.7 (83.3–87.9)	565	92.8 (90.5–94.6)	1346 (88.5)	<0.01 *
k3. The use of objects used by an infected person	672	73.8 (70.8–76.5)	501	82.3 (79.1–85.1)	1173 (77.1)	<0.01 *
k4. Sexual route	281	30.8 (27.9–33.9)	201	33.0 (29.4–36.8)	482 (31.7)	0.399 *
k5. Person-to-person	704	77.3 (74.5–79.9)	479	78.7 (75.3–81.8)	1183 (77.8)	0.571 *
k6. Close contact	632	69.4 (66.3–72.3)	465	76.4 (71.9–79.6)	1097 (72.1)	<0.01 *
Signs and symptoms						
k7. Fever	875	96.0 (94.9–97.2)	587	96.4 (94.7–97.7)	1462 (96.0)	0.786 *
k8. Tiredness	459	50.4 (47.1–53.1)	339	55.7 (51.7–59.6)	798 (52.5)	<0.05 *
k9. Dry cough	780	85.6 (83.2–87.8)	551	90.5 (88.0–92.6)	1331 (87.5)	<0.01 *
k10. Shortness of breath/Breathing difficulties	696	76.4 (73.6–79.1)	544	89.3 (86.7–91.6)	1240 (81.5)	<0.01 *
k11. Aches and pains	433	47.5 (44.3–50.8)	311	51.1 (47.1–55.0)	744 (48.9)	0.191 *
k12. Nasal congestion	261	28.6 (25.8–31.7)	223	36.6 (32.9–40.5)	484 (31.8)	<0.01 *
k13. Running nose	315	34.6 (31.5–37.7)	216	35.5 (31.7–39.3)	531 (35.0)	0.742 *
k14. Sore throat	348	38.2 (35.1–41.4)	326	53.5 (49.6–57.5)	674 (44.3)	<0.01 *
k15. Diarrhea	447	49.1 (47.7–54.2)	335	55.0 (51.0–58.9)	782 (51.4)	<0.05 *
Treatments/prevention						
k16. The incubation period (2 weeks)	786	86.3 (83.9–88.4)	561	92.1 (89.8–94.1)	1347 (88.6)	<0.01 *
k17. COVID-19 vaccines, drugs, or treatments is available	686	75.3 (72.4–78.0)	477	78.3 (74.9–81.5)	1163 (76.5)	0.068 *
k18. Lock-down	475	52.1 (48.9–55.4)	415	68.1 (64.4–71.8)	890 (58.5)	<0.01 *
k19. Self-isolation	417	45.8 (42.6–49.0)	355	58.3 (54.3–62.2)	772 (50.7)	<0.01 *
k20. Home quarantine	770	84.5 (82.1–86.8)	505	82.9 (79.8–85.8)	1275 (83.8)	0.434 *
Summarized knowledge about COVID-19						<0.01 *
Good	226	24.8 (22.1–27.7)	218	35.8 (32.1–39.7)	444 (29.2)	
Poor	685	75.2 (72.3–77.9)	391	64.2 (60.3–67.9)	1076 (70.8)	

All *p*-values are based on a Chi-square test of numbers in urban and rural categories except those indicated by an asterisk (*) which are based on Fisher’s exact test. Scores considered (≥80 = good and <80 = poor).

**Table 3:** Respondents’ attitude towards COVID-19 pandemic among urban and rural respondents of Bangladesh (*n* = 1520).

Attitude Items	Rural		Urban			
	*n*	% (95% C.I)	*n*	% (95% C.I.)	Total *n* (%)	*p*-Value
Total respondents	911	59.9	609	40.1	1520 (100.0)	
A1. The government should lock-down the travel areas to avoid the spread of COVID-19						<0.01
**Strongly agree**	639	70.1 (67.1–73.0)	491	80.6 (77.3–83.6)	1130 (74.3)	
**Agree**	220	24.1 (21.5–27.0)	97	15.9 (13.2–19.0)	317 (20.8)	
Neutral	30	3.3 (2.3–4.6)	10	1.6 (0.8–2.9)	40 (2.6)	
Disagree	19	2.1 (1.3–3.2)	10	1.6 (0.8–2.9)	29 (1.9)	
Strongly disagree	3	0.3 (0.1–0.9)	1	0.2 (0.0–0.8)	4 (0.2)	
A2. Home quarantine can reduce COVID-19 outbreaks						<0.05
**Strongly agree**	449	49.3 (46.0–52.5)	342	56.2 (52.2–60.1)	791 (52.0)	
**Agree**	359	39.4 (36.3–42.6)	216	35.5 (31.7–39.3)	575 (37.8)	
Neutral	48	5.3 (4.0–6.9)	25	4.1 (2.7–5.9)	73 (4.8)	
Disagree	45	4.9 (3.7–6.5)	15	2.5 (1.4–3.9)	60 (3.9)	
Strongly disagree	10	1.1 (0.6–1.9)	11	1.8 (1.0–3.1)	21 (1.3)	
A3. Isolation and treatment of infected people are effective ways to reduce the spread of the virus						<0.01
**Strongly agree**	426	46.8 (43.5–50.0)	355	58.3 (54.3–62.2)	781 (51.3)	
**Agree**	390	42.8 (39.6–46.0)	206	33.8 (30.2–37.7)	596 (39.2)	
Neutral	64	7.0 (5.5–8.8)	39	6.4 (4.7–8.6)	103 (6.7)	
Disagree	27	3.0 (2.0–4.2)	7	1.1 (0.5–2.2)	34 (2.2)	
Strongly disagree	4	0.4 (0.1–1.0)	2	0.3 (.1–1.0)	6 (0.3)	
A4. Personal hygiene is important in controlling the spread of COVID-19						<0.01
**Strongly agree**	545	59.8 (56.6–63.0)	455	74.7 (71.1–78.0)	1000 (65.7)	
**Agree**	315	34.6 (31.5–37.7)	134	24.0 (18.9–25.4)	449 (29.5)	
Neutral	40	4.4 (3.2–5.9)	12	2.0 (1.1–3.3)	52 (3.4)	
Disagree	9	1.0 (0.5–1.8)	7	1.1 (0.5–2.2)	16 (1.0)	
Strongly disagree	2	0.2 (0.0–0.7)	1	0.2 (0.0–0.8)	3 (0.1)	
Summarized attitude towards COVID-19						<0.01 *
Good	683	75.0 (72.1–77.7)	517	84.9 (81.9–87.6)	1200 (78.9)	
Poor	228	25.0 (22.3–27.9)	92	15.1 (12.4–18.1)	320 (21.1)	

All *p*-values are based on a Chi-square test of numbers in urban and rural categories except those indicated by an asterisk (*) which are based on Fisher’s exact test. Responses in bold indicate positive attitude. Scores considered (≥80 = good and <80 = poor).

**Table 4:** Good preventive/perceived practices towards COVID-19 pandemic among urban and rural respondents of Bangladesh (*n* = 1520).

Practice Items	Rural		Urban			
	*n*	% (95% C.I)	*n*	% (95% C.I)	Total *n* (%)	*p*-Value
Total respondents	911	59.9	609	40.1	1520 (100.0)	
Preventive practices						
P1. Practice self-isolation/Home quarantine	777	85.3 (82.9–87.5)	561	92.1 (89.8–94.1)	1338 (88.0)	<0.01*
P2. Ensure sufficient food stock	297	32.3 (29.6–35.7)	211	34.4 (30.9–38.5)	508 (33.4)	0.437 *
P3. Practice respiratory hygiene	507	55.7 (52.4–58.9)	371	60.9 (57.0–64.7)	878 (57.7)	<0.05 *
P4. Wash hand frequently using hand sanitizer	712	78.2 (75.4–80.7)	554	91.0 (88.5–93.1)	1266 (83.2)	<0.01 *
P5. Use face mask	697	76.5 (73.7–79.2)	484	79.5 (76.1–82.5)	1181 (77.6)	0.187 *
P6. Avoid touching nose, mouth and eyes	688	75.5 (72.7–78.2)	501	82.3 (79.1–85.1)	1189 (78.2)	<0.01 *
P7. Maintain social distance (min 1 m)	440	48.3 (45.1–51.5)	418	68.6 (64.9–72.2)	858 (56.4)	<0.01 *
P8. Avoid practice of handshake	446	49.0 (45.7–52.2)	319	52.4 (48.4–56.3)	765 (50.3)	0.209 *
P9. Avoid practice of handshake hug	678	74.4 (71.5–77.2)	474	77.8 (74.4–81.0)	1152 (75.7)	0.143 *
P10. Avoid visit to any public places	413	45.3 (42.1–48.6)	230	37.8 (34.0–41.7)	643 (42.3)	<0.01 *
P11. Avoid contact with infected person	796	87.4 (85.1–88.9)	555	91.1 (88.7–93.2)	1351 (88.8)	<0.05 *
P12. Seek immediate medical attention/treatment regarding primary symptoms	556	61.0 (57.8–64.2)	426	70.0 (66.2–73.5)	982 (64.6)	<0.01*
Summarized preventive practices against COVID-19						<0.01 *
Good	200	22.0 (19.4–24.7)	198	32.5 (28.9–36.3)	398 (26.2)	
Poor	711	78.0 (75.3–80.6)	411	67.5 (63.7–71.1)	1122 (73.8)	

All *p*-values are based on a Chi-square test of numbers in urban and rural categories except those indicated by an asterisk (*) which are based on Fisher’s exact test. Scores considered (≥80 = good and <80 = poor).

## Data Availability

The data presented in this study are available on request from the corresponding author.

## Supplementary Materials

The following are available online at https://www.mdpi.com/2673-3986/2/1/1/s1, Figure S1: Study framework, Table S1: Cronbach’s alpha value estimation. Table S2: Analysis of demographic factors, knowledge and attitudes associated with preventive practices of COVID-19 in Bangladesh. Table S3: Multivariable logistic regression analysis with forward conditional method showing predictors of preventive practices of COVID-19 in Bangladesh.
