# Supplementary material for: COVID-19 Epidemic in Bangladesh among Rural and Urban Residents: An Online Cross-Sectional Survey of Knowledge, Attitudes, and Practices
Source: Epidemiologia (Basel). 2020 Dec 22;2(1):1–13. doi: 10.3390/epidemiologia2010001 (PMC9620879; doi:10.3390/epidemiologia2010001)
Supplement: Supplementary file 1 [file epidemiologia-02-00001-s001.pdf]

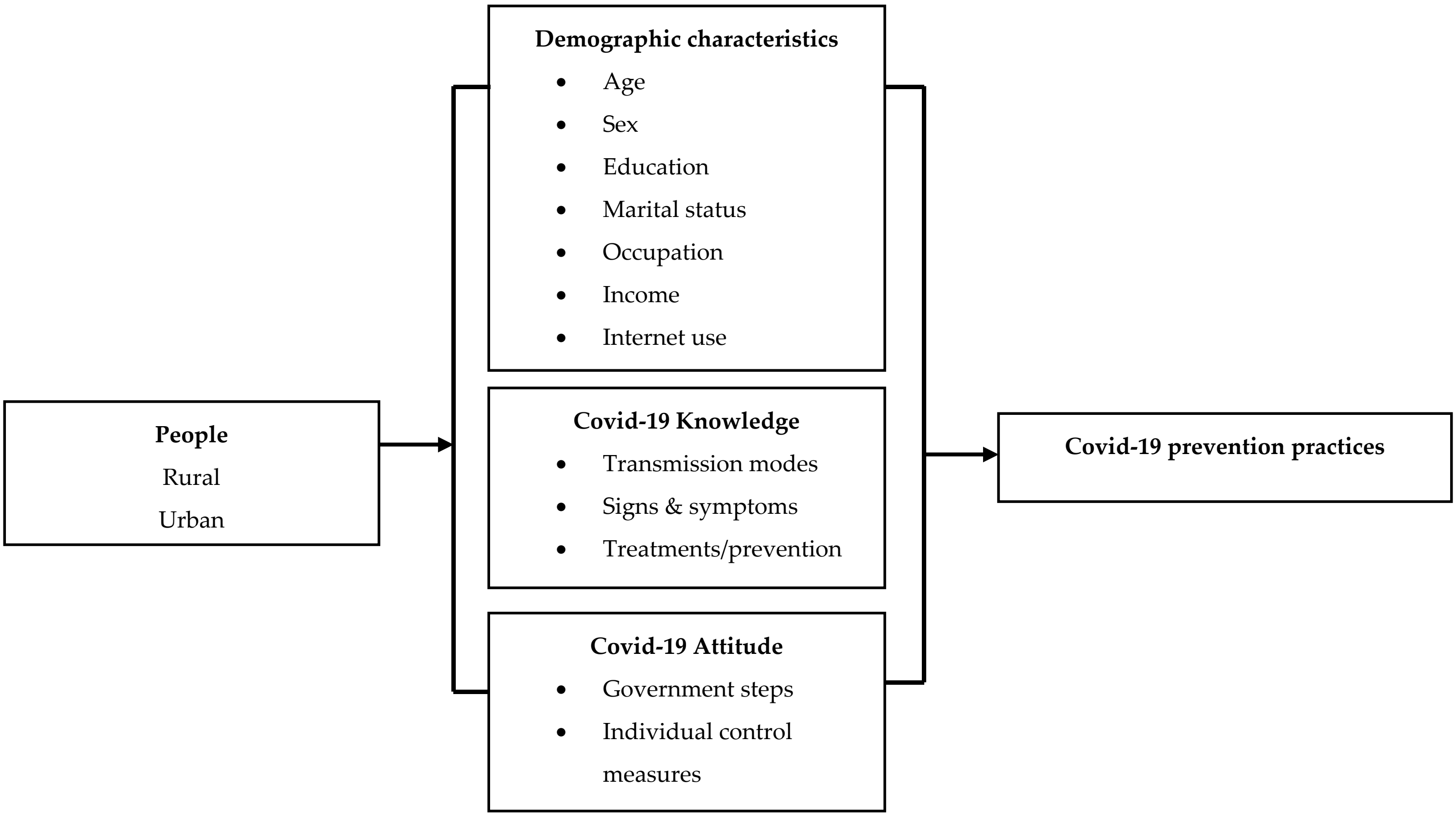

Figure S1. Study framework

Table S1. Cronbach's alpha value estimation.

KAP Items	Reliability
**Knowledge (K1-K20)**	**0.743**
k1	0.728
k2	0.733
k3	0.718
k4	0.728
k5	0.732
k6	0.721
k7	0.740
k8	0.726
k9	0.730
k10	0.725
k11	0.723
k12	0.730
k13	0.717
k14	0.728
k15	0.768
k16	0.791
k17	0.738
k19	0.734
k20	0.747
**Attitude (A1-A4)**	**0.660**
A1	0.618
A2	0.597
A3	0.552
A4	0.605
**Practice (P1-12)**	**0.560**
P1	0.552
P2	0.529
P3	0.518
P4	0.517
P5	0.520
P6	0.502
P7	0.506
P8	0.591
P9	0.566
P10	0.569
P11	0.560
P12	0.617
**Total**	**0.703**

Table S2. Analysis of demographic factors, knowledge and attitudes associated with preventive practices of COVID-19 in Bangladesh.

Variables	Poor practices			
	N	n (%)	OR (95 % C.I)	*P* value
Age group (years)				
<30	725	491 (67.7)	Reference	
30-40	419	300 (71.7)	1.2 (0.9-1.6)	0.172
>40	376	331 (88.0)	3.5 (2.5-4.9)	<0.01
Sex				
Female	575	385 (67.0)	Reference	
Male	945	737 (78.0)	1.7 (1.4-2.2)	<0.01
Education				
> higher secondary	1162	796 (68.5)	Reference	
≤ higher secondary	358	326 (91.1)	4.7 (3.2-6.9)	<0.01
Family Income (Monthly)				
>30000	487	314 (64.5)	Reference	
20000-30000	342	252 (73.7)	1.5 (1.1-2.1)	<0.01
10000-20000	351	281 (80.1)	2.2 (1.6-3.04)	<0.01
<10000	340	275 (80.9)	2.3 (1.7-3.3)	<0.01
Place of residence				
Urban	609	411 (67.5)	Reference	
Rural	911	711 (78.0)	1.7 (1.3-2.2)	<0.01
Use internet to know about COVID-19				
Yes	1356	978 (72.1)	Reference	
No	164	144 (87.8)	2.8 (1.7-4.5)	<0.01
Use television/radio to know about COVID-19				
Yes	989	750 (66.80)	Reference	<0.05
No	531	372 (33.2)	0.7 (0.6-0.9)	
Read newspaper/magazines to know about COVID-19				
Yes	609	406 (36.2)	Reference	
No	911	716 (63.8)	1.8 (1.5-2.3)	<0.01
Use social media to know about COVID-19				
Yes	1148	787 (68.6)	Reference	
No	372	335 (90.1)	4.1 (2.9-5.9)	<0.01
Knowledge of signs & symptoms				
Good	292	165 (56.5)	Reference	
Poor	1228	957 (77.9)	2.7 (2-3.5)	<0.01
Knowledge of transmissions				
Good	666	403 (60.5)	Reference	
Poor	854	719 (84.2)	3.5 (2.7-4.4)	<0.01
Attitudes				
Good	1200	856 (71.3)	Reference	
Poor	320	266 (83.1)	1.9 (1.4-2.7)	<0.01

Scores considered (≥80 = good and <80 = poor). OR odds ratio

Table S3. Multivariable logistic regression analysis with forward conditional method showing predictors of preventive practices of COVID-19 in Bangladesh.

variables	Poor practices			
	N	n (%)	aOR (95 % C.I)	*P* value
Sex				
Female	575	385 (67.0)	Reference	
Male	945	737 (78.0)	1.9 (1.5-2.4)	<0.01
Education				
> higher secondary	1162	796 (68.5)	Reference	
≤ higher secondary	358	326 (91.1)	3.8 (2.6-5.6)	<0.01
Knowledge of transmissions				
Good	666	403 (60.5)	Reference	
Poor	854	719 (84.2)	3.5 (2.7-4.4)	<0.01
Knowledge of signs & symptoms				
Good	292	165 (56.5)	Reference	
Poor	1228	957 (77.9)	1.6 (1.2-2.2)	<0.01
Use television/radio to know about COVID-19				
Yes	989	750 (66.80)	Reference	
No	531	372 (33.2)	0.7 (0.5-0.9)	<0.05
Read newspaper/magazines to know about COVID-19				
Yes	609	406 (36.2)	Reference	<0.01
No	911	716 (63.8)	1.6 (1.2-2)	
Use social media to know about COVID-19				
Yes	1148	787 (68.6)	Reference	
No	372	335 (90.1)	1.9 (1.2-2.9)	<0.01

aOR adjusted odds ratio. Scores considered (≥80 = good and <80 = poor)
